# HIV-1 Nef Inhibits Protease Activity and Its Absence Alters Protein Content of Mature Viral Particles

**DOI:** 10.1371/journal.pone.0095352

**Published:** 2014-04-18

**Authors:** Luiza M. Mendonça, Sandro C. Poeys, Celina M. Abreu, Amilcar Tanuri, Luciana J. Costa

**Affiliations:** 1 Departamento de Virologia, Instituto de Microbiologia Paulo de Góes, Universidade Federal do Rio de Janeiro, Rio de Janeiro, Brazil; 2 Departamento de Genética, Instituto de Biologia, Universidade Federal do Rio de Janeiro, Rio de Janeiro, Rio de Janeiro, Brazil; Lady Davis Institute for Medical Research, Canada

## Abstract

Nef is an important player for viral infectivity and AIDS progression, but the mechanisms involved are not completely understood. It was previously demonstrated that Nef interacts with GagPol through p6*-Protease region. Because p6* and Protease are involved in processing, we explored the effect of Nef on viral Protease activity and virion assembly. Using in vitro assays, we observed that Nef is highly capable of inhibiting Protease activity. The IC50 for *nef*-deficient viruses in drug susceptibility assays were 1.7- to 3.5-fold higher than the wild-type counterpart varying with the type of the Protease inhibitor used. Indicating that, in the absence of Nef, Protease is less sensitive to Protease inhibitors. We compared the protein content between wild-type and *nef*-deficient mature viral particles by gradient sedimentation and observed up to 2.7-fold reduction in the Integrase levels in *nef*-deficient mature particles. This difference in levels of Integrase correlated with the difference in infectivity levels of wild type and *nef*-deficient viral progeny. In addition, an overall decrease in the production of mature particles was detected in *nef*-deficient viruses. Collectively, our data support the hypothesis that the decreased infectivity typical of *nef*-deficient viruses is due to an abnormal function of the viral Protease, which is in turn associated with less mature particles being produced and the loss of Integrase content in these particles, and these results may characterize Nef as a regulator of viral Protease activity.

## Introduction

Nef is a crucial factor for virion infectivity and HIV-1 and SIVmac pathogenesis. The major phenotype associated with Nef expression is the increase in virus infectivity (up to 40-fold when comparing wild-type and *nef*-deleted viruses) [1], [2]. In addition, patients infected with *nef*-deleted viruses seldom progress to AIDS and therefore belong to the 10% of HIV-1 infected patients who are regarded as long-term non-progressors (LTNPs) [3].

The classical functions of Nef involve the downmodulation of several immune receptors (such as CD4, MHC-I and MHC-II), modulation of cellular signaling pathways [4]–[7], as well as modulation of secretory pathways [8]. Additionally, accumulation of reverse transcription products during the initial steps of the viral DNA synthesis only occurs in the presence of Nef [8]–[13]. However, these functions alone cannot explain the increase in viral infectivity due to Nef. For instance, a number of studies now disregard the role of CD4 downmodulation in the viral infectivity increase due to Nef [14]–[16].

Another crucial determinant for high viral infectivity is the correct processing of the precursor polyproteins [17]–[19]. The processing of the structural and enzymatic polyproteins Gag and GagPol is performed by the viral protease (PR), which is itself part of the GagPol precursor protein. The PR activation needs to be precisely regulated to produce fully infectious viral progeny. Changes in both order and moment of cleavages may alter the structure or content of virions, causing them to be noninfectious [20]–[22].

It was previously reported that Nef physically interacts with the p6* region of GagPol, described as having a role in regulating PR activity [23], [24]. In fact, it has been proposed that p6* may act as a zymogen, inhibiting PR activation until the appropriate point in time [25]. Based on this hypothesis, our objective was to investigate the possible impact of Nef on the regulation and processing of PR.

The importance of Nef for pathogenesis and to contribute to an accelerate rate of disease progression is very well established and seems to be related to Nef’s ability to help the virus escape the host immune response. Nonetheless, the role of Nef in generating fully infectious viral progeny may not be related to its influence on the rate of progression toward AIDS because the generation of fully infectious viral progeny is also observed in *in vitro* assays [16]. Because Nef is a multifunctional protein, it is reasonable to hypothesize that these two effects are due to separate functions achieved by Nef interaction with distinct protein partners. The elucidation of the mechanism responsible for the increase in infectivity is of great importance and might culminate in the development of new therapeutics to treat or cure AIDS.

We found that Nef directly inhibits PR activity and that the IC50 values of commercial PR inhibitors when using *nef*-deleted viruses are consistently higher than the values obtained for the WT virus, indicating a diminished PR sensitivity to these drugs and suggesting that PR is overactive in the absence of Nef. This faster processing was associated with loss of Integrase (IN) content in budding particles and with an overall reduction in the production of mature viral particles. Moreover, the reduction in infectivity of *nef*-deficient mature viral particles correlates with the reduction in IN incorporation within these particles. These results can explain the mechanism by which Nef increases the infectivity of the viral progeny, and characterize Nef as an important regulator of PR activity. Thus, Nef can be target for antivirals aiming to disrupt the relationship between Nef and PR, which would have dire consequences to the HIV replicative cycle.

## Materials and Methods

### Antibodies, Antiretroviral Drugs and Vectors

Polyclonal anti-CA, anti-MA and anti-IN were used as primary antibodies for the detection of HIV-1 proteins in WB assays. All these antibodies were obtained through the NIH AIDS Research & Reference Reagents Program. Anti-RT was donated by Dr. Stuart Le Grice, anti-IN by Dr. Duane P. Grandgenett [26] and anti-MA by Dr. Paul Spearman. Primary monoclonal anti-α-tubulin antibody was purchased from Sigma Aldrich, St. Louis, MO. Secondary horseradish peroxidase-conjugated antibodies (GE Healthcare Life Sciences, Fairfield, USA) were also used and were detected by enhanced chemiluminescence (Thermo Fisher Scientific, Rockford, USA). The antiretroviral drugs lopinavir, darunavir, efavirenz and atazanavir sulfate were also obtained through the AIDS Reagent Program, Division of AIDS, NIAID, NIH. The plasmid DNAs encoding replication-competent HIV-1 proviruses were from HIV-1 NL4-3 and NL4-3**Δ**Nef [27] or pBR43eGnef+ and pBR43eGnef− [28], [29].

### Eukaryotic Cells and Transfections

Adherent cell lines Hek-293T and TZM-bl were grown in DMEM supplemented with 10% fetal calf serum and streptomycin-penicillin. Transfections were performed with Fugene 6 (Promega, Madison, WI) according to the manufacturer’s instructions or by using the CaCl_2_ transfection method. The suspension cell line MOLT4 clone 8 was grown in RPMI medium supplemented with 10% fetal calf serum, GlutaMAX (Life Technologies) and streptomycin-penicillin. Transfections were performed by electroporation using the Neon Transfection System (Life Technologies) with the following program: 1400 mV, 3 pulses, 10 ms. When necessary, cell lysates were harvested using RIPA buffer (10 mM Tris-HCl [pH 7.5], 5 mM EDTA, 150 mM NaCl and 0.1% NP-40). The MOLT4 clone 8 and TZM-bl cell lines were obtained through the NIH AIDS Reagent Program, Division of AIDS, NIAID, NIH, from Dr. Ronald Desrosiers [30] and from Dr. John C. Kappes, Dr. Xiaoyun Wu and Tranzyme Inc., respectively.

### Density Gradient Assays

For the sucrose gradient assays, viruses were produced either in Hek-293T cells or MOLT4 clone 8 cells. For the production of viruses in Hek-293T cells, 3×10^6^ cells were transfected with 20 µg of the HIV-1 or HIV-1**Δ**Nef plasmid using the CaCl_2_ transfection method, and the supernatant was harvested 24 hours later. For the sucrose or Iodixanol gradient (Sigma Aldrich, St. Louis, MO) assays using viruses produced in MOLT4 cells, 3×10^6^ cells were electroporated (Neon Transfection System by Invitrogen, Carlsbad, CA) with 30 µg of the HIV-1 or HIV-1**Δ**Nef plasmid, and the supernatant was harvested 24 hours later. To separate the cell-free supernatants containing virus particles, continuous density gradients were prepared by overlaying 2 ml of a 70% sucrose solution prepared in STE buffer (100 mM NaCl, 10 mM Tris pH 8, 1 mM EDTA) with 6 ml of 30% sucrose-STE and letting the gradients rest at 4°C overnight. Filtered cell-free supernatants (3 ml) were loaded on top of the gradient, and the volume was brought up to 12 ml with PBS. The tubes were centrifuged (100,000 g, 16 h, 4°C) in a Beckman SW-41 rotor (Beckman Coulter, Palo Alto, CA). Twelve fractions were collected from the top to bottom of each tube and were assayed using an HIV-1 p24 Antigen ELISA kit (ZeptoMetrix Corporation, Buffalo, USA) and WB. For the WB analyses, the fractions were loaded after protein precipitation with 20% TCA.

The Iodixanol gradients were prepared using the methodology already described [31], with some adaptations. The gradient consisted of eight layers of 1 ml each spanning 9.6% to 18%, and layered with 4 ml of cell free supernatant.

### One-round Drug Susceptibility Assay

For the one-round drug susceptibility assay, viruses were produced by the transfection of MOLT4 or Hek-293T cells with same amounts of the HIV-1 or HIV-1ΔNef plasmids, and the cells were non-treated and treated with increasing concentrations of the corresponding drug: lopinavir, atazanavir, tipranavir and nevirapine for viruses produced in Hek-293T cells and darunavir for viruses produced in MOLT4 cells. For the Hek-293T-produced viruses, 1.25×10^5^ cells were co-transfected with 500 ng of the HIV-1 or HIV-1**Δ**Nef plasmid and 250 ng of a GFP-expressing vector using the Fugene 6 (Promega, Madison, WI) transfection method. Five hours after adding the transfection mix to the cells, medium containing increasing concentrations of lopinavir, atazanavir, tipranavir and nevirapine was added. For the one-round susceptibility assay using viruses produced in MOLT4 cells, 3×10^6^ cells were electroporated (Neon Transfection System by Invitrogen, Carlsbad, CA) with 30 µg of the HIV-1 or HIV-1ΔNef plasmid and 15 µg of a GFP-expressing vector. Six hours later, 2×10^5^ cells were plated in six-well plates, and medium containing increasing concentrations of darunavir was added. At 24 hours p.t., the supernatant and lysate samples were harvested. The supernatants were clarified to remove cell debris by centrifugation at 5,000 g for 3 minutes. An aliquot of supernatants harvested from non-treat NL4-3 and NL4-3ΔNef were used for measurement of p24-CA content by ELISA for in order to confirm equal levels of viral progeny release. Equal volumes of each supernatant were used to infect TZM-bl indicator cell line in order to access viral infectivity. Values of viral infectivity were treated independently for NL4-3 and NL4-3ΔNef, raw values were transformed to percentage of virus infectivity assuming the non-treated condition as 100%. Concentration-response curves and IC50 values were conveniently fitted using Hill 4-parameter non-linear regression. Regression and statistical analyses were performed using GraphPad Prism 6 software (GraphPad Software Inc., La Jolla, CA). *P* values smaller than 0.05 were considered significant. At least three independent experiment for each virus was performed in duplicates.

The remaining supernatant was concentrated by a 16,000 g spin for 2 hours. Three-quarters of the supernatant was discarded carefully from the top of the liquid, and the concentrated supernatant used for the WB analyses. The harvested lysates were also used for WB analyses in order to confirm equivalent levels of protein expression in each condition.

### Kinetic Analyses of PR Activity

To verify the effect of Nef on PR activity *in vitro*, we performed an assay utilizing the clarified lysate of HIV-1 PR-expressing *E. coli* as the source of recombinant PR. Two bacterial clones were used. A Codon Plus *E. coli* (Agilent Technologies, Palo Alto, CA) transformed with the pET11a HIV-1 subtype B PR vector [32] and control non-transformed Codon Plus *E. coli*. A total of 5 ml of pre-inoculum was prepared in LB medium containing 34 µg/ml of chloramphenicol (for both bacteria) and 50 µg/ml ampicillin (only for the pET11a-transformed bacteria). Cultures were grown at 37°C with stirring at 150 rpm for 16 h. After this step, 3 ml of the pre-inoculum was added to 150 ml of LB medium containing the appropriate antibiotics. The OD600 nm was measured over time until it reached 0.7, when 1 mM IPTG was added to the culture to induce protein expression. Two hours later, the bacteria were collected by centrifugation for 30 minutes at 5,000 g in a Sorvall RC-5b centrifuge, lysed by sonication and centrifuged again to separate the clarified supernatant from the cell debris. The clarified supernatant was used in the enzymatic reaction, which was performed using the HIV-1 PR assay kit from ProteinOne. In this assay, we used 20 µl of the clarified lysate and added GST-Nef or GST at two different concentrations (0.5 µM and 0.25 µM) or pepstatin (2 µM). The volume was then brought up to 50 µl using the kit’s Assay Buffer. An aliquot of 50 µl of the specific HIV-1 PR FRET substrate (2 µM) was then added. Upon cleavage, a fluorescent molecule is released, and its concentration can be monitored at excitation/emission wavelengths of 490 nm/530 nm. The fluorescence emission was measured 45 times over a 2-hour period using a Victor X3 fluorimeter (Perkin Elmer, Waltham, MA). A lysate control was also performed using the lysate from non-transfected bacteria treated under the same experimental conditions. All conditions were analyzed in triplicate.

### Densitometry Analyses

Densitometry measurements were performed using the untreated raw scan of the western blots films. The image file was aligned and cropped using Photoshop CS5. For the comparison of different samples from different viruses the membranes were treated in parallel and exposed on the same film using the same procedure and materials (eg. antibody dilutions, ECL reagent), then a single montage containing all the samples was used. Only non-saturated exposures were used for densitometry. At least three different unsaturated exposures were analyzed and the data was used only when the results were reproducible among them. The images were analyzed using Scion Image software (Scion Corporation) using the gelplot2 macro or on ImageJ (NIH) using the Gel function to calculate the area under the curve (AUC) of the band of interest. The values were normalized by fixing the HIV-1 AUC to 1 and calculating the relative fold-difference of HIV-1**Δ**Nef.

### Statistical Analyses

All statistical analyses were performed using GraphPad Prism 6 software (GraphPad Software Inc., La Jolla, CA). Paired Student’s *t* tests were used to assess statistical significance in pairwise comparisons. For analyses of normalized values, one sample *t* tests were performed against a hypothetical value of 1 (representing the HIV-1 reference values). For the one-round drug susceptibility assays the concentration-response curves and IC50 values were fitted using Hill 4-parameter non-linear regression using the normalized infectivity levels as the data source. *p* values smaller than 0.05 were considered significant.

## Results

### A GST-Nef Chimera Directly Inhibits PR Activity *In vitro*

**Figure 1:**
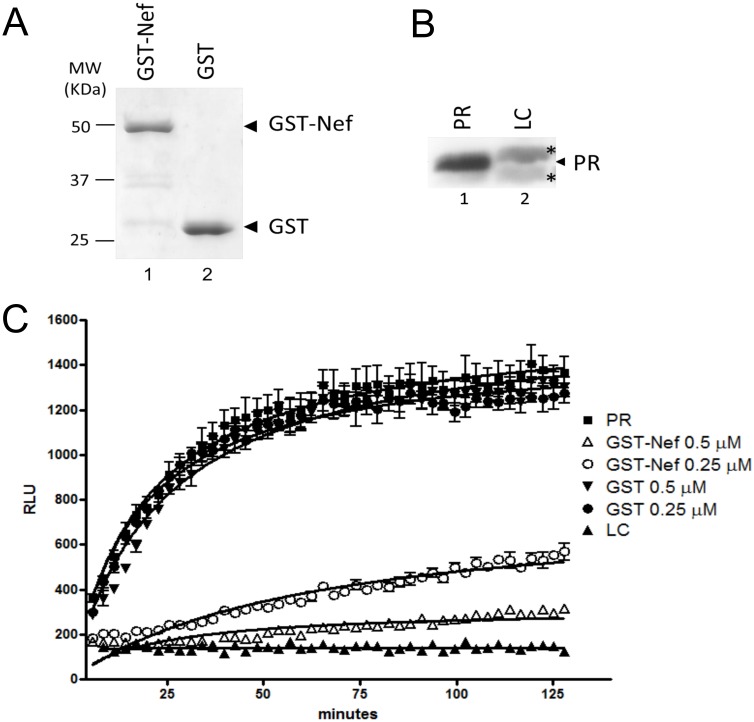
Nef effects in PR activity *in vitro*. (A) SDS-PAGE of purified GST-Nef fusion (lane 1) and GST (lane 2) proteins stained with Coomassie Blue. Molecular weights (MW) are shown on the left. (B) WB of the lysate of *E. coli* expressing HIV-1 Protease (PR) (lane 1) and a lysate control (LC) (lane 2). *Denotes the detection of two nonspecific bands only in the LC. (C) Protease activity measured by the cleavage of a specific FRET substrate over a 2-hour interval. Substrate cleavage allows emission of light and is represented by the y-axis. All conditions were tested in triplicate. RLU – Relative light units.

Nef expression in the producer cell and precise PR regulation are two essential factors for high viral infectivity, however, the direct impact of Nef on PR activity have never been investigated. To assess this possible effect, we performed an *in vitro* kinetic analysis of PR activity in the presence of 0.5 µM or 0.25 µM of a GST-Nef fusion protein or GST as control. The levels and purity of both GST-Nef fusion protein and GST are shown in Figure 1A. The clarified lysate of *E. coli* expressing HIV-1 PR was used as the source of active PR, and the lysate of *E. coli* that expressed no PR was used as control (LC) (Figure 1B). PR activity was assessed by the cleavage of a specific HIV-1 PR substrate consisting of the MA-CA cleavage site conjugated to a FRET fluorophore pair at 2 µM. The aspartyl protease inhibitor Pepstatin (data not shown), and the specific inhibitor of HIV-1 protease saquinavir (Figure S1) were used as controls for HIV-1 PR inhibition. Readings were taken 45 times over a 2-hour period. PR activity was inhibited up to 5-fold using 0.5 µM of GST-Nef fusion protein, which corresponds to 1∶4 molar ratio to the PR substrate, whereas no inhibition was observed in the presence of GST alone (Figure 1C). We also observed that the PR inhibition occurred with a lower concentration (0.25 µM) of the chimeric GST-Nef, albeit resulted in less inhibition of PR activity.

The use of clarified PR-expressing bacterial lysates was due to several unsuccessful attempts to recover an active protease after purification. The fact that PR activity was equal in the presence of substrate alone or in the presence of GST indicates that the PR inhibition in the presence of the GST-Nef chimera was not due to contaminants in the lysate. Moreover, the absence of cleavage when the lysate control was used and the inhibition of PR by the specific inhibitor saquinavir guarantee that the cleavage seen with the clarified lysate of *E. coli* expressing HIV-1 PR is due the specific activity of HIV-1 PR.

These data demonstrate that HIV-1 Nef specifically and directly inhibits PR activity.

### HIV-1ΔNef is Less Sensitive to Protease Inhibitors

**Figure 2:**
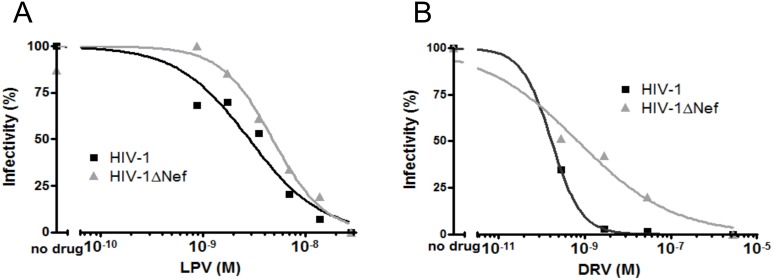
HIV-1ΔNef is less sensitive to Protease Inhibitors. HIV-1 and HIV-1**Δ**Nef viruses were produced in Hek-293T cells treated with increasing concentrations of LPV or in MOLT4 cells treated with increasing concentrations of DRV. The infectivity of the viral progeny was measured in TZM-bl cells, and dose-response curves and IC50 values were fitted using Hill 4-parameter non-linear regression. (A) The concentration-response curves for HIV-1 and HIV-1**Δ**Nef in the presence of LPV. (B) The concentration-response curves for HIV-1 and HIV-1**Δ**Nef in the presence of DRV. Representative of three experiments.

The sensitivity of PR to Protease Inhibitors (PI) is expected to be influenced by any change in the levels of PR activity, and the previous experiment showed Nef alters PR activity. Therefore we sought to evaluate whether Nef+ and Nef− viruses would have different levels of sensitivity to PIs. We performed an one-round drug susceptibility assay with two isogenic molecular clones of HIV-1, NL4-3 (henceforth referred to as HIV-1) and NL**Δ**Nef (henceforth referred to as HIV-1**Δ**Nef). Transfected cells (Hek-293T or MOLT4 clone 8) were maintained in the presence of increasing concentrations of PIs (lopinavir, LPV, or darunavir, DRV) for a 24-hour period, when the viral progeny was harvested and titrated in the TZM-bl indicator cell line. The viral titers were used to calculate the IC50 of each PI for each virus using the Hill 4-parameter non-linear regression. The calculated IC50 values of LPV and DRV for the WT virus were 2.83 nM and 0.17 nM, respectively (Fig. 2A and B and Table 1). For the HIV-1**Δ**Nef virus, the calculated IC50 value of LPV was 2-fold higher (5.84 nM, *p* = 0.005) and that of DRV was 3.5-fold higher (0.6 nM, p<0.001) (Fig. 2 A and B and Table 1).

**Table 1:** Increased concentrations of Protease Inhibitors are required to inhibit HIV-1ΔNef.

Drug	HIV-1 IC50	HIV-1ΔNef IC50	*p*	Class	Cell
Darunavir	0.17 nM	0.60 nM	<0.001	PI	MOLT4
Lopinavir	2.83 nM	5.84 nM	0.005	PI	Hek-293T
Atazanavir	0.28 nM	1.84 nM	0.005	PI	Hek-293T
Tipranavir	6.864 nM	7.638 nM	0.830	PI*	Hek-293T
Nevirapine	114.6 nM	170.8 nM	0.753	NNRTI	Hek-293T

PI – Protease Inhibitor;

NNRTI – Non Nucleosidic Reverse Transcriptase Inhibitor.

*Non-peptidomimetic protease inhibitor.

Western-blotting analyses of the viral progeny released from Hek-293T cells in the presence of lopinavir revealed that less Gag accumulates in HIV-1**Δ**Nef with increasing concentrations of LPV when compared with the wild type HIV-1 (Figure S2). Moreover, the accumulation of Integrase in viral particles also differs in HIV-1 and HIV-1**Δ**Nef viruses, indicating that *nef*-deleted viruses have differences in PI susceptibility compared to wild type viruses.

One-round drug susceptibility assays were also performed using atazanavir, another PI, tipranavir, a non-peptidomimetic PI, and nevirapine, a Non-Nucleoside Reverse Transcriptase Inhibitor (NNRTI), as a control. The IC50 of atazanavir for HIV-1**Δ**Nef was 6.6-fold higher than that for WT HIV-1 (1.84 nM versus 0.58 nM, *p* = 0.0276), indicating that higher concentrations of PIs are required to decrease the infectivity of the *nef*-deficient viruses to 50% than to decrease the infectivity of WT HIV-1 virus. However, there was no difference in the IC50 of tipranavir for HIV and HIV-1**Δ**Nef, suggesting that differences in the mechanism of action of PIs might determine the sensitivity of HIV-1**Δ**Nef to PIs. As expected, the difference in the IC50 of nevirapine between the two viruses was not statistically significant, indicating that this property is restricted to peptidomimetic PIs (Table 1). Altogether, these results indicate that in the absence of Nef the viral PR is less sensitive to peptidomimetic PIs.

### Analyses of Mature Viral Particles in Wild Type and Nef-deleted Viruses

**Figure 3:**
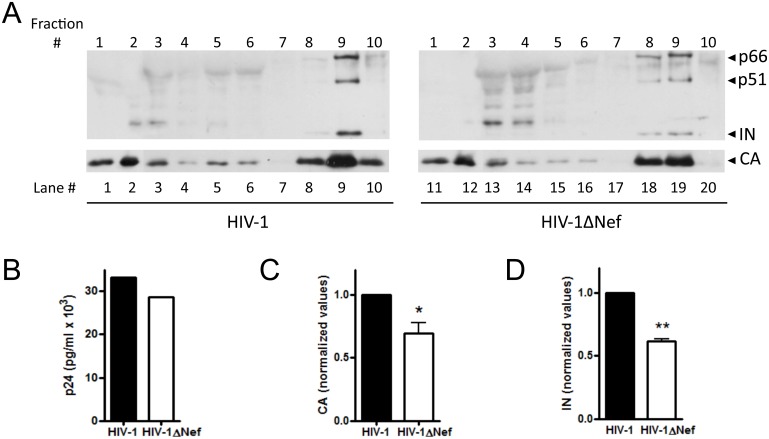
HIV-1 and HIV-1ΔNef mature particles have different protein content and distribution on a density gradient. HIV-1 and HIV-1**Δ**Nef virions were produced in Hek-293T cells and separated using a 30–70% continuous sucrose density gradient. Ten fractions were collected from top to bottom and numbered #1 through #10 accordingly. The fractions were analyzed to determine the content and distribution of IN (top panel) and CA (lower panel). (A) Protein content of soluble proteins (fractions #1–4), immature particles (#5–7) and mature particles (#8–10) for the HIV-1 (left panels) and HIV-1**Δ**Nef viruses (right panels). (B) The CA content of cell-free supernatants before fractionation. (C) Quantification of the amount of CA in fractions #8–10. (D) Quantification of the amount of IN in fraction #8–10. *p = 0.061, **p = 0.036. The WB and ELISA results presented are representative of three experiments, CA and IN values are triplicates.

Previous results pointed out to a possible difference in the processing rate between wild type and *nef*-deleted viruses, which would impact on the quantity and/or quality of released viral progeny. To check for differences between HIV-1 and HIV-**Δ**Nef particles, Hek-293T cells were transfected with HIV-1 and HIV-1**Δ**Nef infectious clones. The culture supernatants were collected 24 h p.t. and directly applied to the top of a 30–70% continuous sucrose density gradient, to allow the analysis of particles in distinct maturation stages. The fractions were analyzed to determine the contents of Reverse Transcriptase (RT), IN and CA. The RT content was used as a marker to identify the fractions that harbored mature viral particles. The first fractions obtained from the top of the gradient (#1 through 4) represent soluble proteins, the intermediate fractions (#5 through 7) represent immature viral particles (5 to 7), and the bottom fractions (#8 through 10) represent mature viral particles. We observed that for the HIV-1 virus, fraction 9 was the peak for mature viral particles, whereas for the HIV-1**Δ**Nef virus, mature particles were distributed between fractions 8 and 9 (Figure 3A, compare lanes 8 through 10 with lanes 18 through 20). The levels of CA in the mature particles differed between the HIV-1 and HIV-1**Δ**Nef viruses, with 30% less CA content on HIV-1**Δ**Nef, *p* = 0.061 (Figs. 3A and C). Importantly, the CA levels in the cell supernatant before fractionation were similar for the two viruses (Fig. 3B), indicating that the observed difference in the protein contents could not be accounted for by differences in particle production. Moreover, a clear difference in the content of IN was observed between the two viruses. The quantification of the IN content of the mature HIV-1 particles also showed 1.6-fold more IN than in the mature HIV-1**Δ**Nef particles, *p* = 0.036 (Figs. 3A and D). These results indicate that the HIV-1**Δ**Nef viral progeny is more heterogeneous, with fewer mature viruses produced and a less IN content relative to HIV-1.

**Figure 4:**
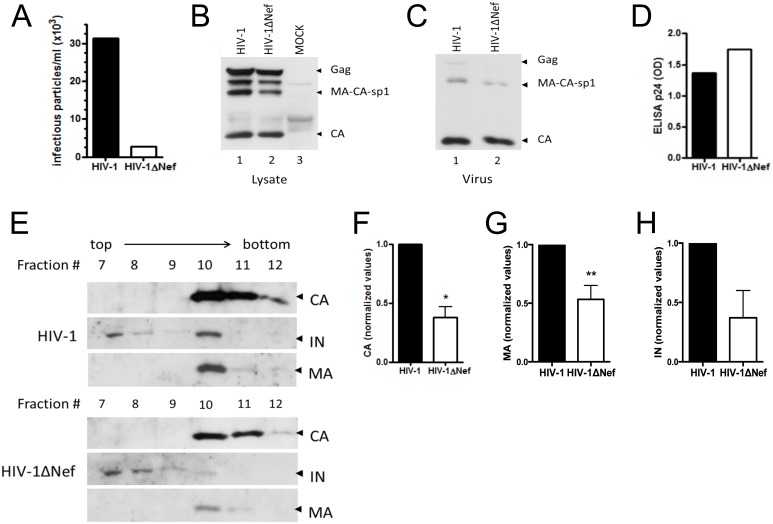
Distribution and protein content differ between HIV-1 and HIV-1ΔNef viral particles produced in MOLT cells. HIV-1 and HIV-1**Δ**Nef virions were produced in MOLT cells and separated using a 30–70% continuous sucrose density gradient. Twelve fractions were collected from top to bottom and numbered #1 through #12 accordingly. The fractions were precipitated with 20% TCA and analyzed by WB. (A) Infectivity levels of the viral progeny produced in MOLT cells. (B) Lysates of HIV-1- and HIV-1**Δ**Nef-transfected cells, showing equivalent levels of viral protein expression. (C) Cell-free supernatants of HIV-1- and HIV-1**Δ**Nef-transfected cells, showing equivalent levels viral release. (D) CA content of cell-free supernatants before separation by the density gradient as measured using a p24-ELISA. (E) CA (top panel), IN (middle pannel) and MA (bottom pannel) protein content of mature particles. The top three panels represent the fractions for HIV-1, and the bottom three panels represent the fractions for HIV-1**Δ**Nef. (F) Quantification of the amount of CA in mature fractions after densitometry, *p = 0.002. (G) Quantification of the amount of MA in mature fractions after densitometry, **p = 0.059 (H) Quantification of the amount of IN in mature particles after densitometry. The results presented are representative of three experiments.

**Figure 5:**
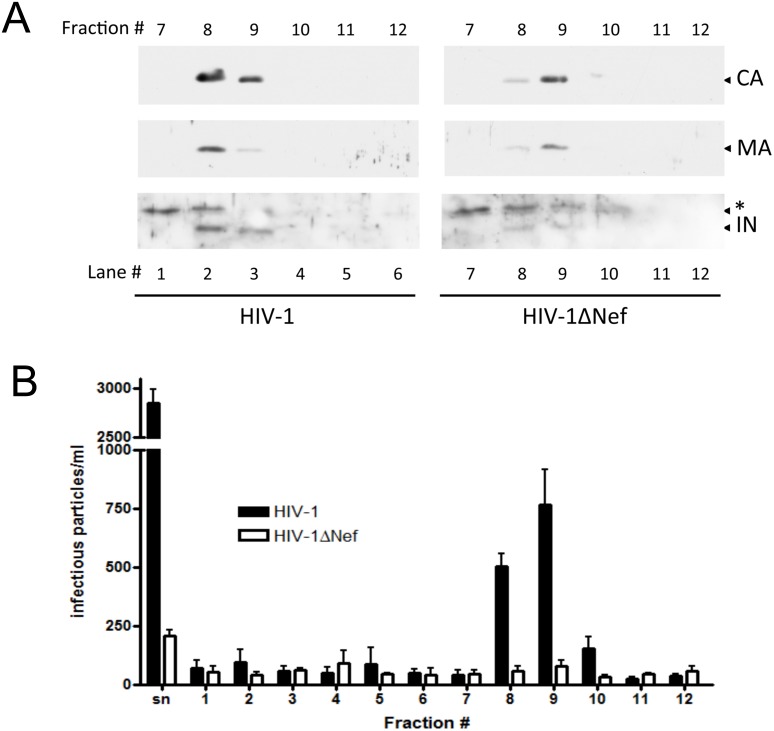
CA, MA and IN levels differ between mature HIV-1 and HIV-1ΔNef viral particles produced in MOLT cells. HIV-1 and HIV-1**Δ**Nef virions were produced in MOLT cells and separated using a 9.6–18% continuous optiprep density gradient. Twelve fractions were collected from top to bottom and numbered #1 through #12 accordingly. The fractions were precipitated with 20% TCA and analyzed by WB to determine the contents and distributions of CA (top panel), MA (middle panel) and IN (bottom panels). Left panels represent the fractions for HIV-1, and the right panels represent the fractions for HIV-1**Δ**Nef. (A) The protein contents of the mature particles for the HIV-1 (left) and HIV-1**Δ**Nef viruses (right). (B) Numbers of blue-foci of non-fractionated supernatant (sn) and non-preciptated fractions for each virus. *Denotes the detection of a nonspecific band. The result presented is the mean of three experiments.

To confirm these results, the same analyses were performed with viral particles produced in a CD4+ lineage using two different types of gradients. The MOLT4 clone 8 cell line is a lymphocytic lineage in which the difference in the infectivity levels of the HIV-1 and HIV-1**Δ**Nef viral progeny varies from 10- to 40-fold (Figure 4A and 5B). MOLT4 cells were electroporated with HIV-1 and HIV-1**Δ**Nef infectious clones. Cell-free supernatants containing viral particles were harvested 24 h p.t. and loaded on the top of a 30–70% continuous sucrose density gradient (Figure 4) or on the top of a 9.6–18% Iodixanol continuous density gradient (Figure 5). Iodixanol gradients exclude vesicles contamination, refining even further our experiments. Cell lysates and cell-free supernatants from the HIV-1 and HIV-1**Δ**Nef viruses were analyzed before fractionation, and these viruses were found to have equivalent levels of viral protein expression (Fig. 4B) and equivalent amounts of viral particle production (Figs. 4C and 4D).

The distribution of mature particles measured by the CA content was equivalent between HIV-1 and HIV-1**Δ**Nef viruses, with particles concentrated in fractions 10 and 11 in the sucrose gradient (Fig. 4E) and fractions 8 and 9 in iodixanol gradients (Fig. 5A). However, the difference in the absolute protein levels was striking. Taking both gradients together, the CA content of HIV-1**Δ**Nef mature viruses was 2.5-fold lower than in HIV-1, *p* = 0.002 (Fig. 4F). Content of MA protein was also altered on HIV-1**Δ**Nef mature viruses, 1.87-fold lower, *p* = 0,059 (Fig. 4G). The IN content was also 2.7-fold higher in the mature HIV-1 particles than in the HIV-1**Δ**Nef mature particles (Fig. 4H). Taken the results of all gradients together, the mean of the difference of IN levels between wild type and nef-deleted viruses after normalization against the difference in the amount of mature particles, given by the CA content, was 2.5-fold (*p* = 0.036, Figure S3). These results confirm the previous results obtained with the viral particles produced in Hek-293T cells; and the discrepancy between the HIV-1 and HIV-1**Δ**Nef protein levels in mature particles was more prominent.

Using Iodixanol gradients, we were able to assess directly the infectivity of viral particles in each fraction. The protein content distribution along the gradient coincides with the distribution of infectious particles for HIV-1 viruses (compare Figs. 5A and 5B). Nonetheless, for HIV-1**Δ**Nef, even though the protein content was concentrated on the same fractions as the HIV-1 infectious viruses were, the number of infectious particles was lower than that of HIV-1. Due to technical constrains we were not able to normalize the viral inoculum against the difference in the amount of CA in fractions 8 and 9 between the two viruses. Therefore the 6.5-fold difference in the number of infectious HIV-1 and HIV-1**Δ**Nef mature particles might be related both to the less amount of these particles being produced and/or the deleterious effect of less amount of IN present in the HIV-1**Δ**Nef particles (Fig. 5B).

These results demonstrate that *nef*-deficient viral particles have deficiencies in maturation and protein content and that this discrepancy is related to the difference in PR activity between the HIV-1 and HIV-1**Δ**Nef viruses and altogether might explain, why *nef*-deficient viruses are less infectious.

## Discussion

In this study, we characterized the ability of Nef to inhibit PR activity *in vitro*. The *nef-*deficient viruses exhibited diminished sensibility to PIs, associated with the reduced production of mature viral particles and to the reduction of IN incorporation into mature viral particles. These differences could account for the decreased infectivity of HIV-1**Δ**Nef viral progeny.

In our assays, we observed that a GST-Nef fusion protein had the specific ability to inhibit HIV-1 PR activity *in vitro*. The GST protein alone had no influence on PR activity, and previous studies have demonstrated that GST proteins, *per se*, do not have inhibitory effects on PR activity [33]. These results suggest that the inhibition observed with the GST-Nef fusion protein is due specifically to Nef. The mechanism by which Nef inhibits PR *in vitro* could be due to competitive inhibition, with Nef functioning as a substrate for PR, as Nef is known to be cleaved by this enzyme [34], [35]. However, a non-competitive model of inhibition cannot be excluded because the FRET substrate was provided at a fourfold to eightfold excess relative to the concentration of the GST-Nef fusion protein. Nonetheless, the observed inhibition with the Nef:substrate ratio used herein was greater than that expected for a competitive inhibition. Moreover, the SQNY/PIV cleavage site present in the FRET substrate is known to be the second most preferred cleavage site in Gag (representing the MA-CA boundary) and has a high affinity constant [36]. The affinity constant of PR for the ACAW/LEAQ cleavage site in Nef has never been characterized.

Our one-round drug susceptibility assay was designed to assess the differences in PR susceptibility to PIs, which are known to be influenced by changes in PR activity, in the presence or absence of Nef. As for the classical phenotyping assays, we can compare the sensitivity of different viruses by comparing the calculated IC50 values for a specific drug. It must be noted that the IC50 values can be compared only with other IC50 values calculated using the same method, as the differences in the nature of the assay do not allow comparisons with IC50 values calculated using classical phenotyping assays, that, for instance, use multiple rounds of infection and use cellular viability as the read-out. In this work, we tested viruses produced in different cell lines in the presence of different PIs. The *nef*-deficient viruses were consistently less sensitive to PIs than the wild-type viruses under all these conditions, thus indicating that either PR is more active in the absence of Nef or that Nef influences PR’s exposure to PIs, making PR more accessible to these drugs. The evidence indicates that protein-protein interactions can alter the accessibility of a substrate to the enzyme [37]. Nonetheless, we found that *nef*-deficient viruses consistently exhibited a higher CA/Gag ratio when compared to wild type viruses (data not shown). The CA/Gag ratio has been classically considered a good indicator of the PR processivity [25], supporting that the PR activity is increased in the absence of Nef. Some mutations in PR result in a slower processing rate in a global manner; these mutations include T26S, G86A, K45I/L90M, K45I/V82S, D30N/V82S and N88D/L90M [38]–[40]. In fact, there is evidence showing that the complementation of a PR-defective infectious clone with a PR harboring the T26S mutation *in trans* mostly overcomes the necessity of Nef for viral infectivity (unpublished data).

Several systems developed for the screening of new antiretroviral compounds use a reporter gene in place of the *nef* ORF to facilitate the read-out [41], [42]. The results presented herein suggest that the absence of Nef expression in these systems will influence the results for compounds targeting PR; therefore, we suggest that, at least for PR inhibitor candidates, other screening systems should be used. Screening assays for IN inhibitors may also take advantage of using *nef* positive reporter constructs, since Nef impacts the amount of incorporated IN within viral particles.

It has been established that the processing of Gag and GagPol does not occur during the early stages of assembly because the premature activation of PR would lead to a loss of protein content in the viral particles [21], [43], [44]. During viral assembly, PR activity is inhibited both by the interaction of p6* in GagPol with the active site of PR [25], [45] and by the oligomerization of Gag and GagPol, which interferes with GagPol dimerization [46]. Nef interacts with GagPol and is a substrate for PR [23], [47]–[50]; therefore, the participation of Nef in this process is possible. Moreover, it has been recently demonstrated that by increasing the amount of Gag at cell membrane and promoting the processing of this polyprotein, Nef would play a role in viral maturation [51].

The results of this study showed that up to 2.7-fold less IN is incorporated into viral particles in the absence of Nef. The boundary between RNaseH and IN is one of the most preferred site cleaved during the processing of GagPol [52]. Premature PR activation can lead to premature processing at this specific cleavage site and would consequently influence the incorporation of IN into viral particles because IN does not possess the proper signals for translocation to the membrane or other ways to attach itself to the site of assembly. This can be a possible explanation for the lower IN content seen in *nef*-deficient particles in this study, however more studies are needed to prove that the increased PR activity is the cause of the lower IN content of *nef*-deficient mature particles.

Several other studies have identified the deleterious effects of increased processing on viral infectivity [53], [54]. The co-transfection of PBMCs with an infectious provirus and a vector that overexpresses PR in a Tat- and Rev-dependent manner led to the accelerated processing of Gag and GagPol, resulting in a 40-fold decrease in viral infectivity [54]. Interestingly, the same reduction in viral infectivity was observed in *nef*-deficient particles produced in PBMCs when compared with wild-type particles [9].

Previous comparisons of the contents of structural and enzymatic protein did not show any difference between wild-type and *nef*-deleted viruses [11], [55]. However, KHAN and colleagues analyzed the protein contents in unfractionated viral particles, whereas FORSHEY & AIKEN compared only the CA contents between the two viruses produced from Hek-293T cells. As demonstrated herein, only after viral particle fractionation is the difference in the CA, MA and IN contents noted because these differences occur specifically in mature viral particles. Most past studies analyzed the contents of particles generated over a period of at least 48 hours after transfection [11], [55]. All the results showed in this study were performed with viruses produced over a 24 hour period, as we saw that the greatest differences between the two viruses could be observed in this time frame. We hypothesize that two are the causes of this phenomenon. First, although the processing speed is higher in the absence of Nef, the amount of precursor available for cleavage is the same, and eventually both viruses reach a plateau where cleavage no longer occurs. Second, shorter expression times allow less saturation of the bands in the WB, improving the visualization of differences and the densitometry analyses.

The codependent incorporation of the viral constituents adds complexity to the interpretation of our data. Once we describe a phenomenon that alters the ratio of proteins in the virus, it is hard to find a viral constituent that can be used as a control for the number of particles being produced. Therefore, the HIV-1**Δ**Nef mature particles may contain fewer structural and enzymatic proteins than wild type mature particles, but the number of particles that achieve maturation can be the same, which would represent a qualitative defect. Another possibility is that the absence of Nef would reduce the number of mature particles produced, which represents a quantitative defect. The lower infectivity found in mature *nef*-deficient particles does not coincide with the lower CA or MA content seen on them. This points out that even though the HIV-1**Δ**Nef viruses have sedimentation rates correspondent to the mature infectious particles, they lack infectivity. This suggests that the defect caused by the absence of Nef, and consequent loss of PR regulation, is not restrained to simply a smaller number of mature particles produced, and that there should be another factor that contributes to the smaller infectivity of HIV-1**Δ**Nef particles. One factor that fits very well in this context is the lower IN content seen on HIV-1**Δ**Nef mature particles. The difference of structural proteins (CA and MA) content on mature particles fractions was about 2-fold between HIV-1 and HIV-1**Δ**Nef. Nonetheless, the difference in infectivity levels was 6.5-fold. This argue that, even if the number of mature particles produced was the same in both viruses, the HIV-1**Δ**Nef mature particles would still be threefold less infectious than wild type mature particles. Suggesting that the absence of Nef causes quantitative and qualitative defects in mature viral particles.

During the viral replicative cycle, the regulation of PR can be the result of a direct interaction between Nef and PR or the synergy of Nef, p6*, PR and other viral or host proteins to modulate PR activity. Although our *in vitro* assay demonstrates that Nef is able to directly inhibit PR activity, there should be a mechanism to relieve Nef’s inhibition and allow proper Gag and GagPol processing. It also remains to be determined whether in the absence of Nef there is an overall increase in the PR processivity rate or the earlier activation of the enzyme. In the latter case, the Nef-PR interaction may interfere with PR-PR dimerization and therefore delay activation until the optimal time point during viral budding.

Our data also reconcile the previously published results demonstrating that although the exogenous RT activity of *nef*-deleted virions does not differ from that of wild-type virions, a deficiency in the accumulation of newly synthesized cDNA in infected cells is observed in the former [9]. It has been demonstrated that IN is a co-factor for reverse transcription [56], [57]. Lower levels of this enzyme in the viral progeny could affect the efficiency of the early steps of reverse transcription in the incoming virus, thus explaining why treatments that induce the natural endogenous reverse transcription (NERT) step can restore the loss of infectivity of *nef*-deleted particles [11].

The results presented herein converge to a model in which Nef acts as a regulator of PR activity, delaying its activation until the appropriate time during the HIV-1 replication cycle. The lack of Nef would therefore cause PR to become overactive, leading to the faster processing of structural and enzymatic viral polyproteins, culminating in abnormalities in enzyme content and the production of fewer mature particles. This model can also perfectly explain why viruses that do not express Nef can be up to 40 fold less infective than their wild-type counterparts in different cell lines. This work shows that the lack of Nef negatively interfere on the viral maturation step in the producer cell line, which will impact on the integration and retrotranscription steps during the infection of the new target cell. In the future, a drug that counteracts Nef would be not only good by its own but it could also synergize with the commercially available RT, PR and IN inhibitors, which can be a great benefit to patients under HAART. In summary, in this work we demonstrated for the first time that a viral accessory protein is directly involved in PR regulation, described a new mechanism of PR regulation and a new function of Nef.

## Supporting Information

Figure S1 **Effect of SQV in PR activity in vitro.** HIV-1 Protease (PR) activity measured by the cleavage of a specific FRET substrate over a 40 minute interval. Increasing concentrations of the Protease Inhibitor Saquinavir (SQV) were added to show specific HIV-1 PR inhibition. Substrate cleavage allows emission of light and is represented by the y-axis. RLU – Relative light units. LC – Lysate Control.
(TIFF)

Figure S2 **HIV-1ΔNef particles show increased Gag processing and altered IN incorporation in the presence of PIs.** Hek-293T cells were transfected with HIV-1 and HIV-1**Δ**Nef infectious clones and treated with increasing concentrations of LPV. Viruses were analyzed for Gag processing and IN content by WB with anti-CA and anti-IN antibodies. (A) Gag processing profile and IN content of HIV-1 (left panel) and HIV-1**Δ**Nef viruses (right panel). Arrows indicate the Gag precursor and the processed MA-CA, CA and IN proteins. (B) The IN levels of each lane. This experiment is representative of three experiments performed.
(TIFF)

Figure S3 **IN content is diminished in HIV-1ΔNef mature particles even after CA normalization.** Quantification of the amount of IN on the mature fractions of all gradients taken together after normalization by the CA content of each fraction. AU – Arbitrary units of the densitometry. SEM – Standard error of the mean.
(TIFF)

## References

[1] Schindler M, Münch J, Brenner M, Stahl-Hennig C, Skowronski J, et al (2004) Comprehensive analysis of nef functions selected in simian immunodeficiency virus-infected macaques. J Virol 78: 10588–10597. 15367626 10.1128/JVI.78.19.10588-10597.2004 PMC516420

[2] Fackler OT, Moris A, Tibroni N, Giese SI, Glass B, et al (2006) Functional characterization of HIV-1 Nef mutants in the context of viral infection. Virology 351: 322–339. 16684552 10.1016/j.virol.2006.03.044

[3] Dyer WB, Ogg GS, Demoitie MA, Jin X, Geczy AF, et al (1999) Strong human immunodeficiency virus (HIV)-specific cytotoxic T-lymphocyte activity in Sydney Blood Bank Cohort patients infected with nef-defective HIV type 1. J Virol 73: 436–443. 9847349 10.1128/jvi.73.1.436-443.1999 PMC103850

[4] Garcia JV, Miller AD (1991) Serine phosphorylation-independent downregulation of cell-surface CD4 by nef. Nature 350: 508–511. 2014052 10.1038/350508a0

[5] Janvier K, Craig H, Le Gall S, Benarous R, Guatelli J, et al (2001) Nef-induced CD4 downregulation: a diacidic sequence in human immunodeficiency virus type 1 Nef does not function as a protein sorting motif through direct binding to beta-COP. J Virol 75: 3971–3976. 11264386 10.1128/JVI.75.8.3971-3976.2001 PMC114888

[6] Geyer M, Fackler OT, Peterlin BM (2001) Structure–function relationships in HIV-1 Nef. EMBO Rep 2: 580–585. 11463741 10.1093/embo-reports/kve141 PMC1083955

[7] Lubben NB, Sahlender DA, Motley AM, Lehner PJ, Benaroch P, et al (2007) HIV-1 Nef-induced down-regulation of MHC class I requires AP-1 and clathrin but not PACS-1 and is impeded by AP-2. Mol Biol Cell 18: 3351–3365. 17581864 10.1091/mbc.E07-03-0218 PMC1951775

[8] Costa LJ, Chen N, Lopes A, Aguiar RS, Tanuri A, et al (2006) Interactions between Nef and AIP1 proliferate multivesicular bodies and facilitate egress of HIV-1. Retrovirology 3: 33. 16764724 10.1186/1742-4690-3-33 PMC1526754

[9] Aiken C, Trono D (1995) Nef stimulates human immunodeficiency virus type 1 proviral DNA synthesis. J Virol 69: 5048–5056. 7541845 10.1128/jvi.69.8.5048-5056.1995 PMC189322

[10] Schwartz O, Maréchal V, Danos O, Heard JM (1995) Human immunodeficiency virus type 1 Nef increases the efficiency of reverse transcription in the infected cell. J Virol 69: 4053–4059. 7539505 10.1128/jvi.69.7.4053-4059.1995 PMC189139

[11] Khan M, Garcia-Barrio M, Powell MD (2001) Restoration of wild-type infectivity to human immunodeficiency virus type 1 strains lacking nef by intravirion reverse transcription. J Virol 75: 12081–12087. 11711598 10.1128/JVI.75.24.12081-12087.2001 PMC116103

[12] Pizzato M, Helander A, Popova E, Calistri A, Zamborlini A, et al (2007) Dynamin 2 is required for the enhancement of HIV-1 infectivity by Nef. Proc Natl Acad Sci U S A 104: 6812–6817. 17412836 10.1073/pnas.0607622104 PMC1871867

[13] Stolp B, Abraham L, Rudolph JM, Fackler OT (2010) Lentiviral Nef proteins utilize PAK2-mediated deregulation of cofilin as a general strategy to interfere with actin remodeling. J Virol 84: 3935–3948. 20147394 10.1128/JVI.02467-09 PMC2849517

[14] Miller MD, Warmerdam MT, Gaston I, Greene WC, Feinberg MB (1994) The human immunodeficiency virus-1 nef gene product: a positive factor for viral infection and replication in primary lymphocytes and macrophages. J Exp Med 179: 101–113. 8270859 10.1084/jem.179.1.101 PMC2191317

[15] Goldsmith MA, Warmerdam MT, Atchison RE, Miller MD, Greene WC (1995) Dissociation of the CD4 downregulation and viral infectivity enhancement functions of human immunodeficiency virus type 1 Nef. J Virol 69: 4112–4121. 7769669 10.1128/jvi.69.7.4112-4121.1995 PMC189146

[16] Chowers MY, Pandori MW, Spina CA, Richman DD, Guatelli JC (1995) The growth advantage conferred by HIV-1 nef is determined at the level of viral DNA formation and is independent of CD4 downregulation. Virology 212: 451–457. 7571414 10.1006/viro.1995.1502

[17] de Marco A, Heuser AM, Glass B, Kräusslich HG, Müller B, et al (2012) Role of the SP2 domain and its proteolytic cleavage in HIV-1 structural maturation and infectivity. J Virol 86: 13708–13716. 23055560 10.1128/JVI.01704-12 PMC3503038

[18] Chiu HC, Wang FD, Chen YM, Wang CT (2006) Effects of human immunodeficiency virus type 1 transframe protein p6* mutations on viral protease-mediated Gag processing. J Gen Virol 87: 2041–2046. 16760407 10.1099/vir.0.81601-0

[19] Adamson CS, Salzwedel K, Freed EO (2009) Virus maturation as a new HIV-1 therapeutic target. Expert Opin Ther Targets 13: 895–908. 19534569 10.1517/14728220903039714 PMC2737327

[20] Kaplan AH, Zack JA, Knigge M, Paul DA, Kempf DJ, et al (1993) Partial inhibition of the human immunodeficiency virus type 1 protease results in aberrant virus assembly and the formation of noninfectious particles. J Virol 67: 4050–4055. 8510215 10.1128/jvi.67.7.4050-4055.1993 PMC237772

[21] Navia MA, McKeever BM (1990) A role for the aspartyl protease from the human immunodeficiency virus type 1 (HIV-1) in the orchestration of virus assembly. Ann N Y Acad Sci 616: 73–85. 2078037 10.1111/j.1749-6632.1990.tb17829.x

[22] Pettit SC, Everitt LE, Choudhury S, Dunn BM, Kaplan AH (2004) Initial cleavage of the human immunodeficiency virus type 1 GagPol precursor by its activated protease occurs by an intramolecular mechanism. J Virol 78: 8477–8485. 15280456 10.1128/JVI.78.16.8477-8485.2004 PMC479095

[23] Costa LJ, Zheng YH, Sabotic J, Mak J, Fackler OT, et al (2004) Nef binds p6* in GagPol during replication of human immunodeficiency virus type 1. J Virol 78: 5311–5323. 15137387 10.1128/JVI.78.10.5311-5323.2004 PMC400368

[24] Ludwig C, Leiherer A, Wagner R (2008) Importance of protease cleavage sites within and flanking human immunodeficiency virus type 1 transframe protein p6* for spatiotemporal regulation of protease activation. J Virol 82: 4573–4584. 18321978 10.1128/JVI.02353-07 PMC2293064

[25] Partin K, Zybarth G, Ehrlich L, DeCrombrugghe M, Wimmer E, et al (1991) Deletion of sequences upstream of the proteinase improves the proteolytic processing of human immunodeficiency virus type 1. Proc Natl Acad Sci U S A 88: 4776–4780. 1647017 10.1073/pnas.88.11.4776 PMC51749

[26] Grandgenett DP, Goodarzi G (1994) Folding of the multidomain human immunodeficiency virus type-I integrase. Protein Sci 3: 888–897. 8069220 10.1002/pro.5560030604 PMC2142885

[27] Adachi A, Gendelman HE, Koenig S, Folks T, Willey R, et al (1986) Production of acquired immunodeficiency syndrome-associated retrovirus in human and nonhuman cells transfected with an infectious molecular clone. J Virol 59: 284–291. 3016298 10.1128/jvi.59.2.284-291.1986 PMC253077

[28] Schindler M, Würfl S, Benaroch P, Greenough TC, Daniels R, et al (2003) Down-modulation of mature major histocompatibility complex class II and up-regulation of invariant chain cell surface expression are well-conserved functions of human and simian immunodeficiency virus nef alleles. J Virol 77: 10548–10556. 12970439 10.1128/JVI.77.19.10548-10556.2003 PMC228419

[29] Schindler M, Münch J, Kutsch O, Li H, Santiago ML, et al (2006) Nef-mediated suppression of T cell activation was lost in a lentiviral lineage that gave rise to HIV-1. Cell 125: 1055–1067. 16777597 10.1016/j.cell.2006.04.033

[30] Kikukawa R, Koyanagi Y, Harada S, Kobayashi N, Hatanaka M, et al (1986) Differential susceptibility to the acquired immunodeficiency syndrome retrovirus in cloned cells of human leukemic T-cell line Molt-4. J Virol 57: 1159–1162. 2419583 10.1128/jvi.57.3.1159-1162.1986 PMC252852

[31] Dettenhofer M, Yu XF (1999) Highly purified human immunodeficiency virus type 1 reveals a virtual absence of Vif in virions. J Virol 73: 1460–1467. 9882352 10.1128/jvi.73.2.1460-1467.1999 PMC103971

[32] Sanches M, Martins NH, Calazans A, Brindeiro ReM, Tanuri A, et al (2004) Crystallization of a non-B and a B mutant HIV protease. Acta Crystallogr D Biol Crystallogr 60: 1625–1627. 15333937 10.1107/S0907444904015276

[33] Kotler M, Simm M, Zhao YS, Sova P, Chao W, et al (1997) Human immunodeficiency virus type 1 (HIV-1) protein Vif inhibits the activity of HIV-1 protease in bacteria and in vitro. J Virol 71: 5774–5781. 9223465 10.1128/jvi.71.8.5774-5781.1997 PMC191831

[34] Miller MD, Warmerdam MT, Ferrell SS, Benitez R, Greene WC (1997) Intravirion generation of the C-terminal core domain of HIV-1 Nef by the HIV-1 protease is insufficient to enhance viral infectivity. Virology 234: 215–225. 9268152 10.1006/viro.1997.8641

[35] Pandori M, Craig H, Moutouh L, Corbeil J, Guatelli J (1998) Virological importance of the protease-cleavage site in human immunodeficiency virus type 1 Nef is independent of both intravirion processing and CD4 down-regulation. Virology 251: 302–316. 9837795 10.1006/viro.1998.9407

[36] Pettit SC, Lindquist JN, Kaplan AH, Swanstrom R (2005) Processing sites in the human immunodeficiency virus type 1 (HIV-1) Gag-Pro-Pol precursor are cleaved by the viral protease at different rates. Retrovirology 2: 66. 16262906 10.1186/1742-4690-2-66 PMC1291402

[37] Wyckoff EE, Lloyd RE, Ehrenfeld E (1992) Relationship of eukaryotic initiation factor 3 to poliovirus-induced p220 cleavage activity. J Virol 66: 2943–2951. 1313911 10.1128/jvi.66.5.2943-2951.1992 PMC241053

[38] Mahalingam B, Louis JM, Hung J, Harrison RW, Weber IT (2001) Structural implications of drug-resistant mutants of HIV-1 protease: high-resolution crystal structures of the mutant protease/substrate analogue complexes. Proteins 43: 455–464. 11340661 10.1002/prot.1057

[39] Ishima R, Gong Q, Tie Y, Weber IT, Louis JM (2010) Highly conserved glycine 86 and arginine 87 residues contribute differently to the structure and activity of the mature HIV-1 protease. Proteins 78: 1015–1025. 19899162 10.1002/prot.22625 PMC2811763

[40] Westerman KA, Ao Z, Cohen EA, Leboulch P (2007) Design of a trans protease lentiviral packaging system that produces high titer virus. Retrovirology 4: 96. 18163907 10.1186/1742-4690-4-96 PMC2259377

[41] Garcia-Perez J, Sanchez-Palomino S, Perez-Olmeda M, Fernandez B, Alcami J (2007) A new strategy based on recombinant viruses as a tool for assessing drug susceptibility of human immunodeficiency virus type 1. J Med Virol 79: 127–137. 17177310 10.1002/jmv.20770

[42] Covens K, Dekeersmaeker N, Schrooten Y, Weber J, Schols D, et al (2009) Novel recombinant virus assay for measuring susceptibility of human immunodeficiency virus type 1 group M subtypes to clinically approved drugs. J Clin Microbiol 47: 2232–2242. 19403770 10.1128/JCM.01739-08 PMC2708496

[43] Kaplan AH, Manchester M, Swanstrom R (1994) The activity of the protease of human immunodeficiency virus type 1 is initiated at the membrane of infected cells before the release of viral proteins and is required for release to occur with maximum efficiency. J Virol 68: 6782–6786. 8084015 10.1128/jvi.68.10.6782-6786.1994 PMC237104

[44] Adamson CS, Freed EO (2007) Human immunodeficiency virus type 1 assembly, release, and maturation. Adv Pharmacol 55: 347–387. 17586320 10.1016/S1054-3589(07)55010-6

[45] Ludwig M, Kümmel C, Schroeder-Printzen I, Ringert RH, Weidner W (1998) Evaluation of seminal plasma parameters in patients with chronic prostatitis or leukocytospermia. Andrologia 30 Suppl 1 41–47. 9629442 10.1111/j.1439-0272.1998.tb02825.x

[46] Gatlin J, Arrigo SJ, Schmidt MG (1998) HIV-1 protease regulation: the role of the major homology region and adjacent C-terminal capsid sequences. J Biomed Sci 5: 305–308. 9691224 10.1007/BF02255863

[47] Freund J, Kellner R, Konvalinka J, Wolber V, Kräusslich HG, et al (1994) A possible regulation of negative factor (Nef) activity of human immunodeficiency virus type 1 by the viral protease. Eur J Biochem 223: 589–593. 8055930 10.1111/j.1432-1033.1994.tb19029.x

[48] Freund J, Kellner R, Houthaeve T, Kalbitzer HR (1994) Stability and proteolytic domains of Nef protein from human immunodeficiency virus (HIV) type 1. Eur J Biochem 221: 811–819. 8174561 10.1111/j.1432-1033.1994.tb18795.x

[49] Gaedigk-Nitschko K, Schön A, Wachinger G, Erfle V, Kohleisen B (1995) Cleavage of recombinant and cell derived human immunodeficiency virus 1 (HIV-1) Nef protein by HIV-1 protease. FEBS Lett 357: 275–278. 7835426 10.1016/0014-5793(94)01370-g

[50] Schorr J, Kellner R, Fackler O, Freund J, Konvalinka J, et al (1996) Specific cleavage sites of Nef proteins from human immunodeficiency virus types 1 and 2 for the viral proteases. J Virol 70: 9051–9054. 8971042 10.1128/jvi.70.12.9051-9054.1996 PMC191010

[51] Malbec M, Sourisseau M, Guivel-Benhassine F, Porrot F, Blanchet F, et al (2013) HIV-1 Nef promotes the localization of Gag to the cell membrane and facilitates viral cell-to-cell transfer. Retrovirology 10: 80. 23899341 10.1186/1742-4690-10-80 PMC3734038

[52] Pettit SC, Clemente JC, Jeung JA, Dunn BM, Kaplan AH (2005) Ordered processing of the human immunodeficiency virus type 1 GagPol precursor is influenced by the context of the embedded viral protease. J Virol 79: 10601–10607. 16051852 10.1128/JVI.79.16.10601-10607.2005 PMC1182631

[53] Karacostas V, Wolffe EJ, Nagashima K, Gonda MA, Moss B (1993) Overexpression of the HIV-1 gag-pol polyprotein results in intracellular activation of HIV-1 protease and inhibition of assembly and budding of virus-like particles. Virology 193: 661–671. 7681610 10.1006/viro.1993.1174

[54] Luukkonen BG, Fenyö EM, Schwartz S (1995) Overexpression of human immunodeficiency virus type 1 protease increases intracellular cleavage of Gag and reduces virus infectivity. Virology 206: 854–865. 7856098 10.1006/viro.1995.1008

[55] Forshey BM, Aiken C (2003) Disassembly of human immunodeficiency virus type 1 cores in vitro reveals association of Nef with the subviral ribonucleoprotein complex. J Virol 77: 4409–4414. 12634398 10.1128/JVI.77.7.4409-4414.2003 PMC150647

[56] Wu X, Liu H, Xiao H, Conway JA, Hehl E, et al (1999) Human immunodeficiency virus type 1 integrase protein promotes reverse transcription through specific interactions with the nucleoprotein reverse transcription complex. J Virol 73: 2126–2135. 9971795 10.1128/jvi.73.3.2126-2135.1999 PMC104457

[57] Dobard CW, Briones MS, Chow SA (2007) Molecular mechanisms by which human immunodeficiency virus type 1 integrase stimulates the early steps of reverse transcription. J Virol 81: 10037–10046. 17626089 10.1128/JVI.00519-07 PMC2045400

